# Role of Electron-Driven Proton-Transfer Processes in the Ultrafast Deactivation of Photoexcited Anionic 8-oxoGuanine-Adenine and 8-oxoGuanine-Cytosine Base Pairs

**DOI:** 10.3390/molecules22010135

**Published:** 2017-01-14

**Authors:** Xiuxiu Wu, Tolga N. V. Karsili, Wolfgang Domcke

**Affiliations:** 1 Department of Chemistry, Technische Universitat Munchen, Lichtenbergstr. 4, Garching D-85747, Germany; wuxiuxiu1988@gmail.com (X.W.); domcke@ch.tum.de (W.D.); 2 Department of Chemistry, Temple University, 130 Beury Hall, 1901 N. 13^th^ St., Philadelphia, PA 19122, USA

**Keywords:** oxidative photochemistry, conical intersections, excited state proton-transfer

## Abstract

It has been reported that 8-oxo-7,8-dihydro-guanosine (8-oxo-G), which is the main product of oxidative damage of DNA, can repair cyclobutane pyrimidine dimer (CPD) lesions when incorporated into DNA or RNA strands in proximity to such lesions. It has therefore been suggested that the 8-oxo-G nucleoside may have been a primordial precursor of present-day flavins in DNA or RNA repair. Because the electron transfer leading to the splitting of a thymine-thymine pair in a CPD lesion occurs in the photoexcited state, a reasonably long excited-state lifetime of 8-oxo-G is required. The neutral (protonated) form of 8-oxo-G exhibits a very short (sub-picosecond) intrinsic excited-state lifetime which is unfavorable for repair. It has therefore been argued that the anionic (deprotonated) form of 8-oxo-G, which exhibits a much longer excited-state lifetime, is more likely to be a suitable cofactor for DNA repair. Herein, we have investigated the exited-state quenching mechanisms in the hydrogen-bonded complexes of deprotonated 8-oxo-G^−^ with adenine (A) and cytosine (C) using ab initio wave-function-based electronic-structure calculations. The calculated reaction paths and potential-energy profiles reveal the existence of barrierless electron-driven inter-base proton-transfer reactions which lead to low-lying S_1_/S_0_ conical intersections. The latter can promote ultrafast excited-state deactivation of the anionic base pairs. While the isolated deprotonated 8-oxo-G^−^ nucleoside may have been an efficient primordial repair cofactor, the excited states of the 8-oxo-G^−^-A and 8-oxo-G^−^-C base pairs are likely too short-lived to be efficient electron-transfer repair agents.

## 1. Introduction

The photoinduced dynamics of biological chromophores have been extensively studied in the past two decades. Within this class of organic chromophore systems, the most notable include DNA and RNA nucleobases [1,2,3,4,5,6,7,8,9,10,11,12,13,14,15], nucleosides [1,16,17,18,19,20,21,22,23,24,25,26], and base pairs [27,28,29,30,31,32,33,34,35,36]. Despite strongly absorbing in the near-UV, DNA and RNA nucleobases exhibit a remarkable degree of photostability, although the generation of photoinduced lesions in DNA strands is not fully suppressed [37,38,39]. It is generally believed that the mechanism of the observed photostability of the building blocks of DNA is ultrafast internal conversion of excited state populations to the electronic ground state with the excess energy being dissipated to the surrounding environment as heat. For the isolated nucleobases, there is consensus that internal conversion is mediated by low-lying conical intersections (CIs) which involve excited singlet states of ππ* and/or nπ* character as well as the S_0_ state and become accessible by out-of-plane deformations of six-membered aromatic rings. These CIs dominate the nonradiative decay of the lowest excited states of cytosine (C) [5,7], uracil (U) [8,12], adenine (A) [9,10,11,13,14], guanine (G) [40,41,42,43], and thymine (T) [15,44,45]. At somewhat elevated excitation energies, CIs arising from so-called πσ* states associated with acidic groups are also known to play a role in the photodynamics of the nucleobases. Apart from direct UV excitation, lesions in DNA are also formed by radical-induced oxidation of DNA—leading to oxidized bases such as 8-oxo-guanine (8-oxo-G). Additional paths for UV-induced DNA damage are the formation of radical species either via dissociation or ionization [46,47].

8-oxo-G is one of the most common lesions found in oxidatively damaged DNA [48,49,50]. The oxidation of G to 8-oxo-G substantially reduces the redox potential and enables it to form base pairs with adenine. This may lead to the replacement of G-C pairs by A-T pairs during replication, which is a mutagenic feature common in many forms of cancer [51,52]. Despite these adverse effects, the lower redox potential of 8-oxo-dG (cf. G) makes it a viable candidate for protecting DNA by scavenging highly oxidizing species such as OH radicals [53]. It has also been demonstrated that 8-oxo-G is capable of repairing lesions of cyclobutane pyrimidine dimers (CPD) [54,55]. In the proposed mechanism, the photoexcited state of 8-oxo-G transfers an electron to the CPD, initiating thereby bond cleavage between the pyrimidine bases. This finding suggests that 8-oxo-G may have played an analogous role to modern flavins in prebiotic redox processes [54], rendering its excited state dynamics of particular interest.

Recently, Kohler, Matsika, and coworkers investigated the ultrafast excited-state dynamics of neutral and anionic 8-oxo-deoxyguanosine (8-oxo-dG) in D_2_O solution with femtosecond transient absorption spectroscopy and ab initio calculations [26]. 8-oxo-dG exists in its anionic (deprotonated) form at pH > 7 [56]. The neutral form was found to deactivate to the electronic ground state in <1 ps, whereas the anionic form exhibits a significantly longer excited-state lifetime of ~43 ps [26]. Correspondingly, the latter shows a significant quantum yield for fluorescence [26]. More recent fluorescence up-conversion and theoretical studies led to the conclusion that neutral 8-oxo-G exhibits an ultrafast radiationless decay via two CIs which are accessible by certain out-of-plane deformations of guanine, whilst the longer lifetime of anionic 8-oxo-G^−^ was attributed to the existence of sizable barriers along the reaction paths connecting the Franck-Condon region to the S_1_/S_0_ CIs [57]. This mechanism was also explored by Changenet-Barret et al. for the neutral form [58]. An alternative interpretation is provided by recent studies by Tuna et al. who performed ab initio calculations of excited-state reaction paths for electron/proton transfer between sugar and base for the neutral and anionic forms of the 8-oxo-dG nucleoside, highlighting a barrierless and therefore efficient electron/proton-transfer radiationless deactivation mechanism in the neutral form, while a barrier was found to exist along this reaction path in the anionic form [23]. This finding provides an alternative explanation for the substantially longer excited-state lifetime of the deprotonated form of the 8-oxo-dG nucleoside compared to the neutral form.

In double-stranded DNA, nucleobases are organized in horizontally oriented hydrogen-bonded base pairs and vertically oriented stacks stabilized by π-π interactions. Both architectural motifs may modify the dynamics of the intrinsic decay paths of the individual nucleobases by providing additional decay channels by which the excited-state populations can evolve. Such modifications have been studied, for example, by Crespo-Hernandez and co-workers who have shown that base stacking of A-T DNA oligomers leads to the formation of intra-strand excimer states with lifetimes of 50–150 ps [59] with additional decay features that are somewhat longer lived [60,61]. Kohler and co-workers recently studied the excited-state dynamics of a π-stacked dinucleotide containing the 8-oxo-G^−^ anion at the 5′-end and neutral A at the 3′-end, using time-resolved transient UV-pump IR-probe spectroscopy. They found that UV excitation of the dinucleotide leads to prompt electron transfer from 8-oxo-G^−^ to the π-stacked A, generating a neutral 8-oxo-G radical and an A radical anion [62,63]. For stacked base pairs, the inter-base hydrogen bonds provide additioinal paths along which coupled electron/proton transfer reactions can occur [64,65].

Sobolewski and Domcke and de Vries and coworkers proposed a photoprotective role of excited-state proton transfer in the G-C Watson-Crick (WC) base pair [27,34,35,66]. In these theoretical and experimental studies, the authors suggested that ultrafast excited-state deactivation occurs by inter-base electron-driven proton transfer (EDPT) from G to C. The ab initio electronic-structure calculations identified a low-lying ^1^ππ* charge-transfer (CT) state (arising via an electron promotion from a G-centered π orbital to a C-centered π* orbital). The CT state drives the transfer of a proton from guanine to cytosine. While the CT state is stabilized by the proton transfer, the ground state is destabilized, which results in a barrierless reaction path leading to a low-energy CI of the S_1_ state with the S_0_ state. These findings are supported by pump-probe experiments in solution [21] as well as by recent transient UV pump and IR probe experiments in the gas phase [22]. The inter-base EDPT reaction was shown to be the main path by which internal conversion to the ground state proceeds in the G-C WC base pair in the gas phase and in the bulk DNA environment [27,29,30,31,32,34,35,36,66]. For the A-T WC base pair, EDPT has also been identified as an efficient deactivation path after photoexcitation by ab initio calculations [28,30,33], although an experimental verification of the predicted ultrashort lifetime of the A-T WC base pair is still lacking. 

**Figure 1:**
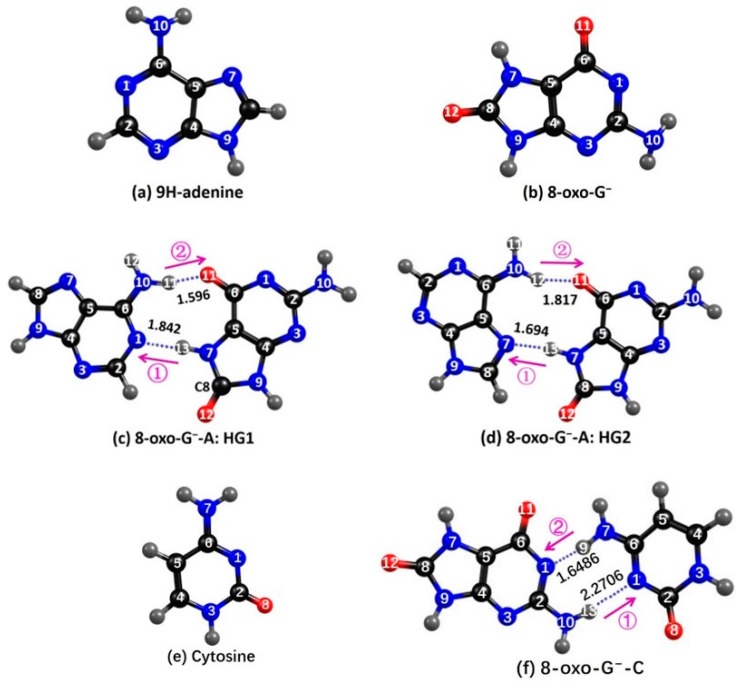
Ground-state equilibrium geometry of (**a**) isolated 9*H*-adenine; (**b**) isolated 8-oxo-G^−^; (**c**) HG1 form of 8-oxo-G^—^A; (**d**) HG2 form of 8-oxo-G^—^A; (**e**) isolated cytosine; and (**f**) 8-oxo-G^−^-C.

8-oxo-G^−^ can pair with A via Hoogsteen (HG) base pairing in two low-energy conformations [67]: HG1 and HG2 (see Figure 1). 8-oxo-G^−^ can also form a stable pair with cytosine in a structure involving two hydrogen bonds, see Figure 1f. EDPT processes in the neutral 8-oxo-G-A and 8-oxo-G-C base pairs were investigated by Kumar and Sevilla with time-dependent density functional (TD-DFT) calculations [67]. In the present work, we focus on EDPT reactions in the anionic 8-oxo-G^−^-A and 8-oxo-G^−^-C base pairs. Since anionic 8-oxo-G^−^ has been shown to have a substantially longer excited-state lifetime than neutral 8-oxo-G, the former appears better suited for light-driven DNA repair reactions than the short-lived neutral 8-oxo-G. It is therefore of interest to explore how base-pairing with A or C affects the excited-state lifetime of anionic 8-oxo-G^−^. As well as base pairing with cytosine (i.e., the complementary base to G), 8-oxoG^−^ is also well-known to form mismatched base pairing with A via a Hoogsteen configuration [68,69,70]. This propensity for forming Hoogsteen base pairs with adenine is due to the enhanced redox potential of 8-oxoG^−^ compared with that of natural guanine.

Using ab initio wave-function based electronic-structure calculations, we identify the EDPT reaction paths leading to CIs through which the excited-state population can internally convert to S_0_. Our findings provide evidence for barrierless EDPT reaction paths and therefore likely highly efficient excited-state deactivation of the 8-oxo-G^−^-A and 8-oxo-G^−^-C base pairs. The efficient excited-state deactivation of the base pairs enhances their photostability, but inevitably also lowers their repair efficiency.

## 2. Results

### 2.1. Ground State Geometries

Figure 1 presents the MP2/cc-pVDZ-optimized ground-state structures of 9*H*-adenine (**a**); 8-oxo-G^−^ (**b**); HG1 (**c**) and HG2 (**d**) 8-oxo-G^−^-A conformers; cytosine (**e**); and the 8-oxo-G^−^-C base pair (**f**). In both 8-oxo-G^−^-A HG base pairs, all atoms are in a common plane with the exception of the wagging angle of the amino group of 8-oxo-G^−^. Adenine retains a planar geometry since the amino group of adenine is involved in the inter-base hydrogen bonding, while in the structure of isolated adenine there is some pyramidization of the amino group.

In the HG1 and HG2 8-oxo-G^−^-A base pairs, 8-oxo-G^−^ and A act both as hydrogen-bond donors and as hydrogen-bond acceptors. There are two hydrogen bonds in the HG1 base pair: N10−H11(A)•••O11(8-oxo-G^−^) and N7−H13(8-oxo-G^−^)•••N1(A). The HG2 base pair also has also two hydrogen bonds, N10−H12(A)•••O11(8-oxo-G^−^) and N7−H13(8-oxo-G^−^)•••N7(A). The calculated hydrogen-bond lengths are included in Figure 1. In the HG1 base pair, the length of N10−H11•••O11(1.596 Å) is shorter than that of N7−H13•••N1(1.842 Å). In contrast, in the HG2 base pair, N10−H12•••O11(1.817 Å) is longer than N7−H13•••N7(1.694 Å). The optimized ground-state energy of the HG2 base pair is found to be lower than that of the HG1 base pair by 0.05 eV, which indicates a Boltzmann population of 13:87 for HG1:HG2. For the 8-oxo-G^−^-C base pair, three low-energy H-bonded configurations have been optimized. Among these, the structure shown in Figure 1f is the lowest-energy conformer.

### 2.2. Vertical Excitation Energies

**Figure 2:**
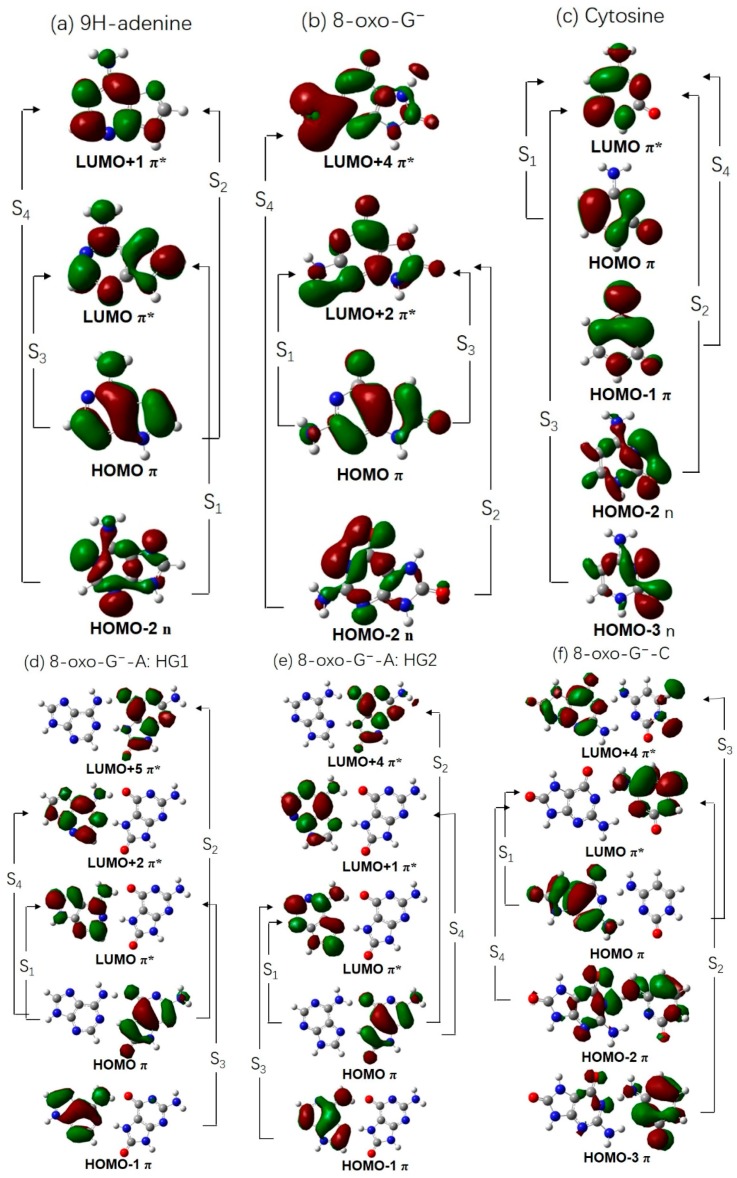
Orbitals and orbital promotions involved in forming the lowest four excited states of 9*H*-adenine (**a**), 8-oxo-G^−^ (**b**), cytosine (**c**), and the three base pairs (**d**–**f**).

Table 1 lists the calculated vertical excitation energies and corresponding oscillator strengths (in parentheses) of the lowest four singlet excited states of the 8-oxoG^−^ containing base pairs presently studied. For comparison, the analogous vertical excitation energies of isolated cytosine, 9*H*-adenine and 8-oxo-G^−^ are presented in Table 2. In addition to Table 1, the reader is directed to Figure 2, which depicts the orbitals and orbital promotions associated with the formation of the lowest four excited electronic states of isolated 9*H*-adenine, cytosine, 8-oxo-G^−^, as well as the 8-oxo-G^−^-A and 8-oxo-G^−^-C base pairs.

As is well known, the lowest four excited states of 9*H*-adenine are of ^1^nπ*, ^1^ππ*(L_b_), ^1^ππ*(L_a_), and ^1^nπ* character. The S_1_ and S_4_ states involve promotion of an electron from an in-plane nitrogen 2p_y_ orbital to a ring-centered π* orbital, while the S_2_ and S_3_ states involve π*←π orbital promotions which are delocalized over the aromatic rings. For 8-oxo-G^−^, the lowest four excited states are ^1^ππ*(S_1_), ^1^nπ*(S_2_), ^1^ππ*(S_3_), and ^1^nπ*(S_4_) in nature. As shown in Figure 2, the S_2_ and S_4_ states involve the promotion of an electron from the nitrogen 2p_y_ orbital to a ring-centered anti-bonding π* orbital. The S_1_ and S_3_ states involve excitation from a ring-centered π orbital to the lowest π* orbital. 

In the two 8-oxo-G^−^-A HG base pairs, the orbital promotions are almost the same and the lowest four excited states are of ^1^ππ* character. The S_1_ state involves electron promotion from the ring-centered π HOMO localized on 8-oxo-G^−^ to the ring-centered π* LUMO localized on adenine, leading to a charge-separated state of CT character. The S_2_ state involves a π*←π promotion, whereby both orbitals are localized on 8-oxo-G^−^. The S_3_ state involves a π*←π promotion localized on adenine. The S_2_ and S_3_ states are therefore locally-excited (LE) states on 8-oxo-G^−^ and A, respectively. The vertical excitation energies of the S_2_ and S_3_ states are comparable to that of isolated 8-oxo-G^−^ and adenine, respectively. As can be seen in Table 1, the vertical excitation energy of the S_2_ (^1^ππ*) state of the HG1/HG2 base pair (4.85 eV/4.89 eV) is nearly equal to that of the analogous LE state (i.e., the first ^1^ππ* state) of isolated 8-oxo-G^−^ (4.92 eV), suggesting that pairing of 8-oxo-G^−^ with adenine has little effect on the lowest LE ^1^ππ* state energy. In contrast, the vertical excitation energies of all electronic states show a significant red-shift upon complexation relative to that of isolated adenine. Similar to the S_1_ state, the S_4_ state also is of CT character, involving the transition from a π orbital of 8-oxo-G^−^ to an π* orbital of adenine, with a much higher excitation energy. The vertical excitation energies of the two HG base pairs are very similar and lower than those of isolated adenine and 8-oxo-G^−^. Compared with the TD-DFT results for neutral 8-oxo-G-A HG base pair [67], the lowest ^1^ππ* transition localized on 8-oxo-G^−^ is blue-shifted, while the lowest ^1^ππ* transition localized on adenine is red-shifted.

Figure 2f depicts the orbital promotions associated with the 8-oxoG^−^-C base pair. As shown, the electronic excitation to S_1_ involves a π to π* electron promotion in which the former is localized on the 8-oxoG^−^ moiety, whereas the latter is localized on the C moiety. As with the 8-oxo-G^−^-A base pairs, electronic excitation to S_1_ involves a significant charge separation and is thus of CT character. In contrast, electronic excitation to the S_2_, S_3_, and S_4_ states involves electron promotions between π/n to π* orbitals that are localized on the same nucleobase within the base pair. The observed orbital ordering is very similar to that of the WC-type G-C base pair which also has an S_1_ state of CT character, whilst the higher-lying states are of LE character [34,35].

### 2.3. Electron-Driven Proton-Transfer Decay Paths

#### 2.3.1. 8-oxoG^−^-A

**Figure 3:**
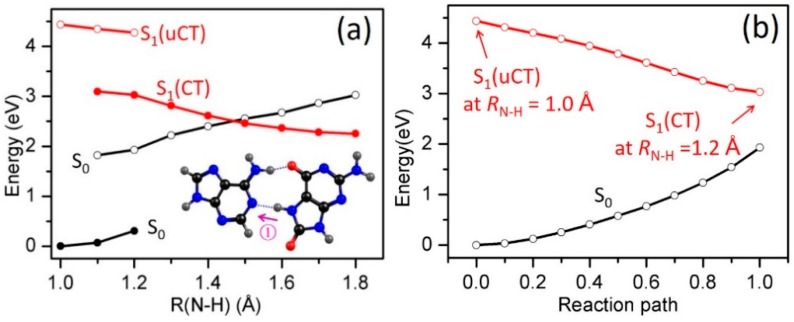

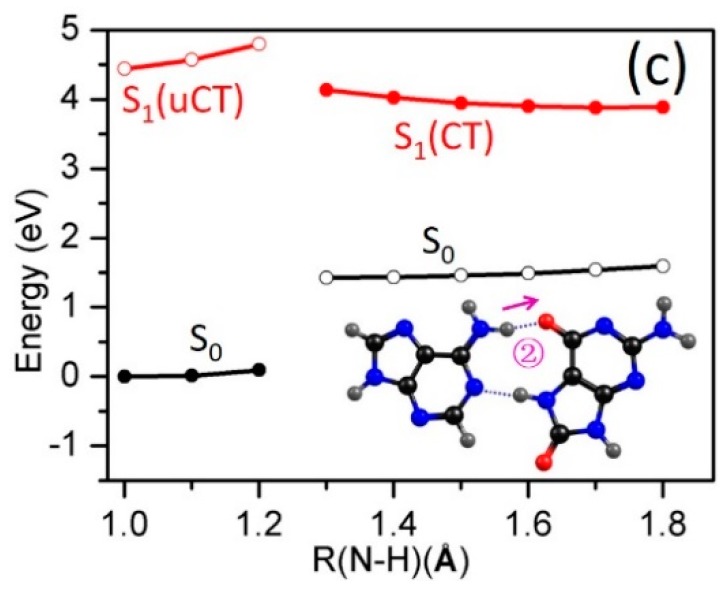
PE profiles of the ground state and the lowest singlet excited states of the HG1 8-oxo-G^−^-A base pair along *R*_N7−H13_ (**a**) and *R*_N10−H11_ (**c**) proton-transfer coordinates; (**b**) shows the energy profiles of the LIIC path connecting S_1_(uCT) with S_1_(CT) in (**a**).

**Figure 4:**
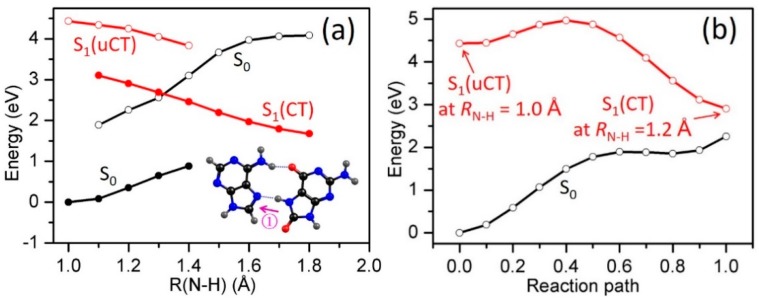

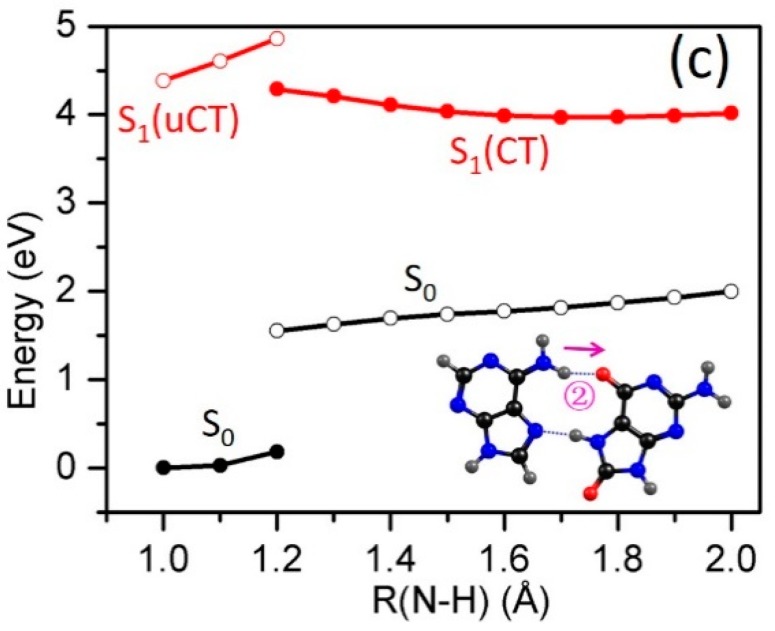
PE profiles of the ground state and the lowest singlet excited states of the HG2 8-oxo-G^−^-A base pair along the *R*_N7−H13_ (**a**) and the *R*_N10−H12_ (**c**) proton-transfer coordinates; (**b**) shows the energy profiles of the LIIC path connecting S_1_(uCT) with S_1_(CT) in (**a**).

In order to study the intrinsic photophysical properties of the two HG base pairs, we explored the details of the potential-energy (PE) profiles along possible inter-base electron and proton transfer paths. The HG1 and HG2 base pairs have two potential reaction paths for proton transfer, one involving the transfer of a proton from adenine to 8-oxo-G^−^ along the N10−H11•••O11 or N10−H12•••O11 hydrogen bonds, the other involving the transfer of a proton from 8-oxo-G^−^ to adenine along the N7−H13•••N1 or N7−H13•••N7 hydrogen bonds (as indicated by the arrows in Figure 1c,d). In order to study the energetics associated with a particular excited-state electron/proton transfer reaction, we computed the PE profiles along the *R*_N10−H11_, *R*_N7−H13_ bond-stretching coordinates for the HG1 base pair and along the *R*_N10−H12_, *R*_N7−H13_ bond-stretching coordinates for the HG2 base pair. The results are depicted in Figure 3 and Figure 4, respectively. In these figures, the filled black circles represent the S_0_ energy profile calculated along the reaction path optimized in the S_0_ state for the specific *R*_N−H_ driving coordinate. The energy of the unrelaxed ^1^ππ* CT state (henceforth ^1^ππ*(uCT)) of the base pairs, calculated at the S_0_-relaxed geometries, is designated by the profile plotted with the open red circles. The filled red circles represent the energy of the lowest inter-base CT state along the proton-transfer relaxed scan optimized for this state. The curve plotted with open black circles gives the energy of the S_0_ state calculated along the minimum-energy reaction path determined in the CT state.

Figure 3a shows the PE profiles associated with proton transfer along N7−H13•••N1 (Path 1, see inset in Figure 3a) in the HG1 base pair. The S_0_ energies calculated along the reaction path relaxed in the S_0_ state rise steadily upon *R*_N7__−H13_ bond extension, showing that proton transfer is unfavorable in this electronic state. When the energy of the ^1^ππ*(uCT) state is optimized for fixed *R*_N7__−H13_ = 1.2 Å, the electronic character of this state changes from LE character to CT character, which implies the transfer of an electron localized on 8-oxo-G^−^ to the π* orbital localized on 9*H*-adenine, resulting in an electronic charge separation. The path connecting the ^1^ππ*(uCT) state at *R*_N7−H13_ = 1.0 Å to the ^1^ππ*(CT) state at its optimized geometry for *R*_N7__−H13_ = 1.2 Å was constructed as a linearly interpolated reaction path. The corresponding energy profile is shown in Figure 3b. This energy profile exhibits no barrier, which ensures that the minimum-energy path connecting the ^1^ππ*(uCT) and ^1^ππ*(CT) states is barrierless. The relaxed ^1^ππ*(CT) profile (Figure 3a, full red circles) shows a strong decrease of the energy as a function of *R*_N7__−H13_ stretching; this represents the driving force towards proton transfer that results from the charge-separated character of the ^1^ππ*(CT) state, which is characteristic of EDPT [71]. The S_0_ energy computed at the ^1^ππ*(CT)-relaxed geometries (Figure 3a, black open circles) increases as a function of the *R*_N7__−H13_ stretching coordinate. As a result, the energies of the ^1^ππ*(CT) state and the S_0_ state cross at *R*_N7__−H13_ ≈ 1.45 Å. This S_1_/S_0_ crossing becomes a CI when the appropriate coupling modes are taken into account. Depending on the topography of the PE surfaces and the nonadiabatic coupling at the CI, the reaction can lead to internal conversion to the S_0_ state of the complex (adiabatic path) or to a biradical.

The other possible pathway by which inter-base proton transfer can occur in the HG1 base pair is along the N10−H11•••O11 hydrogen bond (Path 2, see inset in Figure 3c). Figure 3c shows the PE profiles of the S_0_, ^1^ππ*(uCT) and ^1^ππ*(CT) states along the *R*_N10−H11_ driving coordinate. The LIIC path connecting the ^1^ππ*(uCT) and ^1^ππ*(CT) states (not shown) exhibits no barrier. As for Path 1, the energy profile along the minimum-energy path from the ^1^ππ*(uCT) state to the ^1^ππ*(CT) state is barrierless. However, the ^1^ππ*(uCT) and ^1^ππ*(CT) energies do not cross along *R*_N10−H11_ (Figure 3c), in contrast to the energies along *R*_N7−H13_. This result can easily be rationalized. While the electron transfer occurs from 8-oxo-G^−^ to A, the proton has to move in the opposite direction, from A to 8-oxo-G^−^, which is not energetically favorable. The S_1_(CT) state is therefore not stabilized by the transfer of the proton and the EDPT mechanism does not apply for Path 2 in the HG1 base pair. 

The PE profiles of the lowest excited states of ^1^ππ* (uCT) and ^1^ππ* (CT) character of the HG2 base pair as a function of the *R*_N7−H13_ are shown in Figure 4a. In the HG2 base pair, there likewise exists a proton-transfer path (N7−H13•••N7), which leads to a low-lying S_1_/S_0_ CI, and a proton-transfer path (N10−H12•••O11) which does not lead to a CI. The mechanistic details of the N7−H13•••N7 reaction path (Path 1, see inset in Figure 4a) are similar to those described for the Path 1 in the HG1 base pair. The relaxed ^1^ππ* (CT) state exhibits a distinct driving force for proton transfer and its energy crosses the S_0_ energy along this path at *R*_N7−H13_ ≈ 1.30 Å, giving rise to a CI, representing a route by which either ultrafast IC to the ground state or biradical formation can occur. The linearly interpolated reaction path connecting the ^1^ππ* (uCT) and ^1^ππ* (CT) states is shown in Figure 4b. The energy profile exhibits a substantial barrier of approximately 0.5 eV, which represents an upper limit to the reaction barrier along the minimum-energy path. The access of the photoexcited HG2 8-oxo-G^−^-A base pair to the S_1_/S_0_ CI may thus be kinetically hindered.

The proton-transfer path along the *R*_N10−H11_ driving coordinate in the HG2 base pair is similar to Path 2 in the HG1 base pair. Figure 4c shows the PE profiles of the S_0_, ^1^ππ*(uCT) and ^1^ππ*(CT) states along the *R*_N10−H11_ driving coordinate. While the LIIC path connecting the ^1^ππ*(uCT) and ^1^ππ*(CT) states (not shown) exhibits no barrier, the ^1^ππ*(uCT) and ^1^ππ*(CT) energies do not exhibit a crossing along *R*_N10−H11_, as is shown in Figure 3c. As in the HG1 base pair, the Coulomb attraction after electron transfer from 8-oxo-G^−^ to adenine renders the proton transfer from 8-oxo-G to the adenine anion (Path 1) favorable, while it renders the proton transfer from the adenine anion to 8-oxo-G^−^ (Path 2) unfavorable.

#### 2.3.2. 8-oxo-G^−^-C

**Figure 5:**
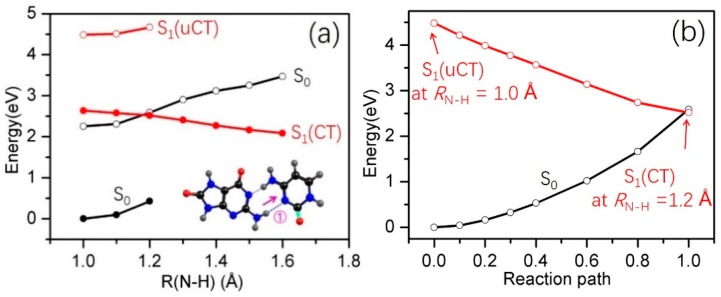

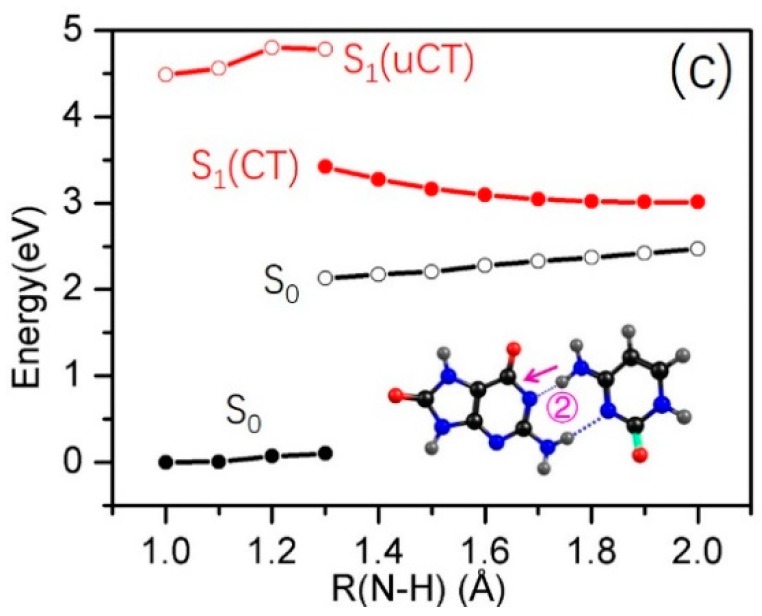
PE profiles of the ground state and the lowest excited states of the 8-oxo-G^−^-C base pair along the *R*_N10−H13_ (**a**) and *R*_N7−H9_ (**c**) proton-transfer coordinates; (**b**) shows the energy profiles of the LIIC path connecting S_1_(uCT) with S_1_(CT) in (**a**).

We now turn our attention to the 8-oxoG^−^-C base pair.The PE profiles along the *R*_N-H_ driving coordinate are depicted in Figure 5. As with 8-oxoG^−^-A, the base pair under consideration exhibits two possible inter-molecular proton-transfer paths along hydrogen bonds as depicted in the insets in Figure 5a,c. Path 1, which involves PT from the N-H donor group of 8-oxo-G^−^ to the N acceptor group of C, shows a barrierless profile with respect to EDPT on S_1_ (Figure 5a). Along this coordinate, the decreasing energy of the S_1_ state is accompanied by an increasing energy of the S_0_ state, which leads to an S_1_/S_0_ curve crossing at *R*_O-H_ ≈ 1.2 Å. At this crossing, the excited-state population can return to the S_0_ state—providing enhanced photostability of the 8-oxoG^−^-C base pair—or a radical pair can be formed. The energy profiles along the LIIC path connecting the ^1^ππ*(uCT) state to the ^1^ππ*(CT) state are shown in Figure 5b. This energy profile exhibits no barrier and leads in fact to an S_1_/S_0_ energy crossing. This result ensures that the minimum-energy path connecting the ^1^ππ*(uCT) and ^1^ππ*(CT) states is barrierless and that the S_1_/S_0_ crossing seam is easily accessible from the Franck-Condon region of the S_1_(uCT) state.

An S_1_/S_0_ crossing is not observed for the second possible proton-transfer path (energy profiles depicted in Figure 5c), although the overall gradients of the S_0_ and S_1_ energy profiles mimick those observed in Figure 5a. The respective decrease and increase of the energies of S_1_(CT) and S_0_ are too weak to lead to a degeneracy of the S_1_ and S_0_ energies. As in the HG1 and HG2 base pairs of 8-oxo-G^−^-A, there exists no substantial driving force for EDPT from cytosine to the 8-oxo-guanine anion.

## 3. General Discussions and Conclusions

We explored the excited-state reaction paths and PE profiles associated with coupled electron/proton transfer reactions in the two most stable hydrogen-bonded conformers of the 8-oxo-G^−^-A base pair as well as in the lowest-energy conformer of the 8-oxo-G^−^-C base pair. In both cases, the 8-oxo-G moiety was assumed to be in its deprotonated (anionic) form which is found in aqueous solution at pH > 7. In the 8-oxo-G^−^-A HG1 base pair as well as in the 8-oxo-G^−^-C base pair, the calculated PE profiles reveal the existence of a barrierless path for EDPT from 8-oxo-G^−^ to A or C, leading to a low-lying S_1_/S_0_ conical intersection which can promote ultrafast excited-state deactivation. In the 8-oxo-G^−^-A HG2 base pair, on the other hand, a low barrier may exist on the S_1_ PE surface which may possibly kinetically hinder the access of this conformer to the S_1_/S_0_ CI. We did not find evidence for the existence of S_1_/S_0_ conical intersections along reaction paths for proton transfer from adenine or cytosine to the 8-oxo-G^−^ anion in any of the three base pairs. The EDPT reactions revealed in the present work for the 8-oxo-G^−^-A HG1 and 8-oxo-G^−^-C base pairs are rather similar to those identified earlier in the G-C and A-T WC base pairs [33,34].

These results are of relevance for the current discussion on the potential role of 8-oxo-G as a photo-repair agent in DNA, possibly being a precursor of modern flavine cofactors [54,55,72]. It is firmly established that the photo-excited state of neutral 8-oxo-G has a sub-picosecond lifetime in aqueous solution, while deprotonated 8-oxo-G^−^ exhibits a much longer fluorescence lifetime of 43 ps [26]. The drastic shortening of the excited-state lifetime of 8-oxo-G^−^ relative to neutral 8-oxo-G has been explained by either CIs intrinsic to guanine, which are more easily accessible in the neutral than in the anionic form [57], or by an EDPT reaction along the H-bond between guanine and ribose in 8-oxo-guanosine, which is available in the neutral form, but not in the anionic form [23]. It has been speculated that the long lifetime of anionic 8-oxo-G^−^ should be favorable for repair by electron transfer in the excited state, while the very short excited-state lifetime of neutral 8-oxo-G should be detrimental in this respect [26]. Herein, we have found computational evidence for presumably very efficient excited-state deactivation via barrierless EDPT reactions leading to S_1_/S_0_ conical intersections in the 8-oxo-G^−^-A and 8-oxo-G^−^G base pairs which call the concept of repair of CPD lesions via electron transfer from excited-state 8-oxo-G^−^ in DNA oligomers into question. Kumar and Sevilla investigated the corresponding EDPT paths in the neutral 8-oxo-G-A and 8-oxo-G-C base pairs and found a path with a barrierless PE profile en route to a low-lying S_1_/S_0_ conical intersection in the 8-oxo-G-C base pair, while no such path was found for the 8-oxo-G-A base pair [67]. This finding led Kumar and Sevilla to the conclusion that the 8-oxo-G-A base-pair, due to its longer excited-state life time, should allow for efficient repair of CPD lesions. However, the very short intrinsic lifetime of neutral 8-oxo-guanosine, not considered by Kumar and Sevilla, renders it unlikely that the neutral 8-oxo-G-A base pairs are efficient repair agents in DNA oligomers. Notwithstanding, we do however stress that isolated nucleobasic or nucleosidic forms of 8-oxoG- may be efficient at repairing CPD lesions—as advocated by Matsika and co-workers [26] and Tuna et al. [23].

There exist additional complexities in a bulk DNA environment which are not taken into account in the present calculations. Electrostatic and dispersive interactions between stacked DNA bases may modify the topographies of the PE profiles and the locations and energies of CIs. Nonetheless, the present calculations for isolated base pairs are useful as they can serve as a starting point for forthcoming studies which include the effect of complex environments, albeit at a more approximate level of theory. 

## 4. Computational Methods

The ground-state minimum-energy geometries of the 8-oxo-G^−^-A base pairs, in the two HG conformations, and of the 8-oxo-G^−^-C base pair were optimized at the MP2/cc-pVDZ level of theory [73,74]. At these ground-state minimum-energy geometries, the vertical excitation energies and oscillator strengths of the lowest four singlet excited states were calculated using the ADC(2) method. [75]. In the MP2 and ADC(2) calculations, the resolution of the identity (RI) approximation was employed in the evaluation of the electron repulsion integrals [76].

The reaction path for inter-base hydrogen-atom transfer from 8-oxo-G^−^ to adenine in the electronic ground state was calculated as a relaxed scan at the MP2 level using *R*_N-H_ of the hydrogen-bonded NH group of 8-oxo-G^−^ as the driving coordinate. This involves scanning of the appropriate *R*_N-H_ driving coordinate, while allowing the rest of the nuclear framework to relax. The energies of the ^1^ππ* excited states along the relaxed ground-state path were computed using the ADC(2) method. Relaxed scans along *R*_N-H_ were also computed for the lowest excited state of charge-transfer (CT) character using the ADC(2) method. In these calculations, the energies of the electronic ground state and the uCT ^1^ππ* states were computed at the relaxed geometries of the CT state using the MP2 and ADC(2) methods, respectively. When calculations of relaxed scans were not possible due to failure of excited-state geometry optimization, an approximate reaction path was constructed by linear interpolation in internal coordinates (LIIC). All calculations were carried out with Turbomole [77]. 

## Figures and Tables

**Table 1:** Vertical excitation energies (∆*E*) and oscillator strengths (*f*) of the lowest four excited states of the two 8-oxo-G^−^-A HG base pairs and the 8-oxo-G^−^-C base pair, calculated at the ADC(2)/cc-pVDZ level of theory. Here O^−^ stands for 8-oxo-G^−^.

State	∆*E*/eV (*f*)	State	∆*E*/eV (*f*)	State	∆*E*/eV (*f*)
8-oxo-G^−^-A HG1		8-oxo-G^−^-A HG2		8-oxo-G^−^-C	
S_1_^1^ππ*(O^−^→A)	4.44 (0.0139)	S_1_^1^ππ*(O^−^→A)	4.39 (0.0091)	S_1_^1^ππ*(O^−^→C)	4.48 (0.0023)
S_2_^1^ππ*(O^−^→O^−^)	4.85 (0.2321)	S_2_^1^ππ*(O^−^→O^−^)	4.89 (0.1025)	S_2_^1^ππ*(C→C)	4.76 (0.0176)
S_3_^1^ππ*(A→A)	4.91 (0.1050)	S_3_^1^ππ*(A→A)	4.95 (0.0922)	S_3_^1^ππ*(O^−^→O^−^)	4.88 (0.0695)
S_4_^1^ππ*(O^−^→A)	5.09 (0.0109)	S_4_^1^ππ*(O^−^→A)	5.18 (0.0281)	S_4_^1^ππ*(O^−^→C)	4.94 (0.0782)

**Table 2:** Vertical excitation energies *(∆E*) and oscillator strengths (*f*) of the lowest four excited states of isolated 9*H*-adenine, anionic 8-oxo-guanine, and cytosine, calculated at the ADC(2)/cc-pVDZ level of theory.

State	∆*E*/eV (*f*)	State	∆*E*/eV (*f*)	State	∆*E*/eV (*f*)
9*H*-adenine		8-oxo-G^−^		Cytosine	
S_1_^1^nπ*	5.13 (0.0051)	S_1_^1^ππ*	4.92 (0.0629)	S_1_^1^ππ*	4.65 (0.0545)
S_2_^1^ππ*	5.27 (0.0152)	S_2_^1^nπ*	5.16 (0.0000)	S_2_^1^nπ*	4.81 (0.0019)
S_3_^1^ππ*	5.40 (0.2856)	S_3_^1^ππ*	5.47 (0.2964)	S_3_^1^nπ*	5.29 (0.0016)
S_4_^1^nπ*	5.82 (0.0018)	S_4_^1^nπ*	5.54 (0.0003)	S_4_^1^ππ*	5.76 (0.1261)

## References

[1] Pecourt J.-M.L., Peon J., Kohler B. (2001). DNA excited-state dynamics: Ultrafast internal conversion and vibrational cooling in a series of nucleosides. J. Am. Chem. Soc..

[2] Kim N.J., Jeong G., Kim Y.S., Sung J., Keun Kim S., Park Y.D. (2000). Resonant two-photon ionization and laser induced fluorescence spectroscopy of jet-cooled adenine. J. Chem. Phys..

[3] Nir E., Kleinermanns K., Grace L., de Vries M.S. (2001). On the photochemistry of purine nucleobases. J. Phys. Chem. A.

[4] Canuel C., Mons M., Piuzzi F., Tardivel B., Dimicoli I., Elhanine M. (2005). Excited states dynamics of DNA and RNA bases: Characterization of a stepwise deactivation pathway in the gas phase. J. Chem. Phys..

[5] Ismail N., Blancafort L., Olivucci M., Kohler B., Robb M.A. (2002). Ultrafast decay of electronically excited singlet cytosine via a π,π* to no,π* state switch. J. Am. Chem. Soc..

[6] Sobolewski A., Domcke W. (2002). On the mechanism of nonradiative decay of DNA bases: ab initio and tddft results for the excited states of 9*H*-adenine. Eur. Phys. J. D.

[7] Merchán M., Serrano-Andrés L. (2003). Ultrafast internal conversion of excited cytosine via the lowest ππ* electronic singlet state. J. Am. Chem. Soc..

[8] Matsika S. (2004). Radiationless decay of excited states of uracil through conical intersections. J. Phys. Chem. A.

[9] Perun S., Sobolewski A.L., Domcke W. (2005). Ab initio studies on the radiationless decay mechanisms of the lowest excited singlet states of 9*H*-adenine. J. Am. Chem. Soc..

[10] Perun S., Sobolewski A., Domcke W. (2005). Photostability of 9*H*-adenine: Mechanisms of the radiationless deactivation of the lowest excited singlet states. Chem. Phys..

[11] Marian C.M. (2005). A new pathway for the rapid decay of electronically excited adenine. J. Chem. Phys..

[12] Zgierski M.Z., Patchkovskii S., Fujiwara T., Lim E.C. (2005). On the origin of the ultrafast internal conversion of electronically excited pyrimidine bases. J. Phys. Chem. A.

[13] Chen H., Li S. (2005). Theoretical study toward understanding ultrafast internal conversion of excited 9*H*-adenine. J. Phys. Chem. A.

[14] Blancafort L. (2006). Excited-State potential energy surface for the photophysics of adenine. J. Am. Chem. Soc..

[15] Perun S., Sobolewski A.L., Domcke W. (2006). Conical intersections in thymine. J. Phys. Chem. A.

[16] Buchner F., Ritze H.-H., Lahl J., Lübcke A. (2013). Time-Resolved photoelectron spectroscopy of adenine and adenosine in aqueous solution. Phys. Chem. Chem. Phys..

[17] Camillis S.D., Miles J., Alexander G., Ghafur O., Williams I.D., Townsend D., Greenwood J.B. (2015). Ultrafast non-radiative decay of gas-phase nucleosides. Phys. Chem. Chem. Phys..

[18] Gustavsson T., Sarkar N., Vaya I., Jimenez M.C., Markovitsi D., Improta R. (2013). A joint experimental/theoretical study of the ultrafast excited state deactivation of deoxyadenosine and 9-methyladenine in water and acetonitrile. Photochem. Photobiol. Sci..

[19] Pecourt J.-M.L., Peon J., Kohler B. (2000). Ultrafast internal conversion of electronically excited RNA and DNA nucleosides in water. J. Am. Chem. Soc..

[20] Peon J., Zewail A.H. (2001). DNA/RNA nucleotides and nucleosides: Direct measurement of excited-state lifetimes by femtosecond fluorescence up-conversion. Chem. Phys. Lett..

[21] Schwalb N.K., Temps F. (2007). Ultrafast electronic relaxation in guanosine is promoted by hydrogen bonding with cytidine. J. Am. Chem. Soc..

[22] Stavros V.G., Verlet J.R. (2016). Gas-Phase femtosecond particle spectroscopy: A bottom-up approach to nucleotide dynamics. Annu. Rev. Phys. Chem..

[23] Tuna D., Domcke W. (2016). Excited-State deactivation in 8-oxo-deoxyguanosine: Comparison between anionic and neutral forms. Phys. Chem. Chem. Phys..

[24] Tuna D., Sobolewski A.L., Domcke W. (2013). Mechanisms of ultrafast excited-state deactivation in adenosine. J. Phys. Chem. A.

[25] Zgierski M.Z., Alavi S. (2006). Quantum chemical study of biradical decay channels in cytidine nucleosides. Chem. Phys. Lett..

[26] Zhang Y., Dood J., Beckstead A., Chen J., Li X.-B., Burrows C.J., Lu Z., Matsika S., Kohler B. (2013). Ultrafast excited-state dynamics and vibrational cooling of 8-oxo-7,8-dihydro-2′-deoxyguanosine in D_2_O. J. Phys. Chem. A.

[27] Abo-Riziq A., Grace L., Nir E., Kabelac M., Hobza P., de Vries M.S. (2005). Photochemical selectivity in guanine-cytosine base-pair structures. Proc. Natl. Acad. Sci. USA.

[28] Gobbo J.P., Saurí V., Roca-Sanjuán D., Serrano-Andrés L., Merchán M., Borin A.C. (2012). On the deactivation mechanisms of adenine-thymine base pair. J. Phys. Chem. B.

[29] Groenhof G., Schäfer L.V., Boggio-Pasqua M., Goette M., Grubmüller H., Robb M.A. (2007). Ultrafast deactivation of an excited cytosine−guanine base pair in DNA. J. Am. Chem. Soc..

[30] Marchetti B., Karsili T.N.V., Ashfold M.N.R., Domcke W. (2016). A ‘bottom up’, ab initio computational approach to understanding fundamental photophysical processes in nitrogen containing heterocycles, DNA bases and base pairs. Phys. Chem. Chem. Phys..

[31] Markwick P.R.L., Doltsinis N.L. (2007). Ultrafast repair of irradiated DNA: Nonadiabatic ab initio simulations of the guanine-cytosine photocycle. J. Chem. Phys..

[32] Nir E., Plützer C., Kleinermanns K., de Vries M. (2002). Properties of isolated DNA bases, base pairs and nucleosides examined by laser spectroscopy. Eur. Phys. J. D.

[33] Perun S., Sobolewski A.L., Domcke W. (2006). Role of electron-driven proton-transfer processes in the excited-state deactivation of the adenine-thymine base pair. J. Phys. Chem. A.

[34] Sobolewski A.L., Domcke W. (2004). Ab initio studies on the photophysics of the guanine-cytosine base pair. Phys. Chem. Chem. Phys..

[35] Sobolewski A.L., Domcke W., Hättig C. (2005). Tautomeric selectivity of the excited-state lifetime of guanine/cytosine base pairs: The role of electron-driven proton-transfer processes. Proc. Natl. Acad. Sci. USA.

[36] Yamazaki S., Taketsugu T. (2012). Photoreaction channels of the guanine-cytosine base pair explored by long-range corrected TDDFT calculations. Phys. Chem. Chem. Phys..

[37] Taylor J.S. (1994). Unraveling the molecular pathway from sunlight to skin cancer. Acc. Chem. Res..

[38] Sinha R.P., Häder D.-P. (2002). UV-Induced DNA damage and repair: A review. Photochem. Photobiol. Sci..

[39] Schreier W.J., Gilch P., Zinth W. (2015). Early events of DNA photodamage. Annu. Rev. Phys. Chem..

[40] Chen H., Li S. (2006). Ab initio study on deactivation pathways of excited 9*H*-guanine. J. Chem. Phys..

[41] Marian C.M. (2007). The guanine tautomer puzzle: Quantum chemical investigation of ground and excited states. J. Phys. Chem. A.

[42] Serrano-Andres L., Merchan M., Borin A.C. (2008). A three-state model for the photophysics of guanine. J. Am. Chem. Soc..

[43] Yamazaki S., Domcke W. (2008). Ab initio studies on the photophysics of guanine tautomers: Out-of-plane deformation and NH dissociation pathways to conical intersections. J. Phys. Chem. A.

[44] Hudock H.R., Levine B.G., Thompson A.L., Satzger H., Townsend D., Gador N., Ullrich S., Stolow A., Martínez T.J. (2007). Ab initio molecular dynamics and time-resolved photoelectron spectroscopy of electronically excited uracil and thymine. J. Phys. Chem. A.

[45] Zechmann G., Barbatti M. (2008). Photophysics and deactivation pathways of thymine. J. Phys. Chem. A.

[46] Gomez-Mendoza M., Banyasz A., Douki T., Markovitsi D., Ravanat J.-L. (2016). Direct oxidative damage of naked DNA generated upon absorption of UV radiation by nucleobases. J. Phys. Chem. Lett..

[47] Banyasz A., Martinez-Fernandez L., Ketola T.-M., Muñoz-Losa A., Esposito L., Markovitsi D., Improta R. (2016). Excited state pathways leading to formation of adenine dimers. J. Phys. Chem. Lett..

[48] Cadet J., Douki T., Ravanat J.-L. (2008). Oxidatively generated damage to the guanine moiety of DNA: Mechanistic aspects and formation in cells. Acc. Chem. Res..

[49] Kanvah S., Joseph J., Schuster G.B., Barnett R.N., Cleveland C.L., Landman U. (2010). Oxidation of DNA: Damage to nucleobases. Acc. Chem. Res..

[50] Markus T.Z., Daube S.S., Naaman R., Fleming A.M., Muller J.G., Burrows C.J. (2009). Electronic structure of DNA-unique properties of 8-oxoguanosine. J. Am. Chem. Soc..

[51] Shibutani S., Takeshita M., Grollman A.P. (1991). Insertion of specific bases during DNA synthesis past the oxidation-damaged base 8-oxodG. Nature.

[52] Greenman C., Stephens P., Smith R., Dalgliesh G.L., Hunter C., Bignell G., Davies H., Teague J., Butler A., Stevens C. (2007). Patterns of somatic mutation in human cancer genomes. Nature.

[53] Steenken S., Jovanovic S.V., Bietti M., Bernhard K. (2000). The trap depth (in DNA) of 8-oxo-7,8-dihydro-2′deoxyguanosine as derived from electron-transfer equilibria in aqueous solution. J. Am. Chem. Soc..

[54] Nguyen K.V., Burrows C.J. (2011). A prebiotic role for 8-oxoguanosine as a flavin mimic in pyrimidine dimer photorepair. J. Am. Chem. Soc..

[55] Nguyen K.V., Burrows C.J. (2012). Photorepair of cyclobutane pyrimidine dimers by 8-oxopurine nucleosides. J. Phys. Org. Chem..

[56] Jayanth N., Ramachandran S., Puranik M. (2009). Solution structure of the DNA damage lesion 8-oxoguanosine from ultraviolet resonance Raman spectroscopy. J. Phys. Chem. A.

[57] Lu Z., Beckstead A.A., Kohler B., Matsika S. (2015). Excited state relaxation of neutral and basic 8-oxoguanine. J. Phys. Chem. B.

[58] Changenet-Barret P., Gustavsson T., Improta R., Markovitsi D. (2015). Ultrafast excited-state deactivation of 8-hydroxy-2′-deoxyguanosine studied by femtosecond fluorescence spectroscopy and quantum-chemical calculations. J. Phys. Chem. A.

[59] Crespo-Hernandez C.E., Cohen B., Kohler B. (2005). Base stacking controls excited-state dynamics in A-T DNA. Nature.

[60] Vayá I., Gustavsson T., Douki T., Berlin Y., Markovitsi D. (2012). Electronic excitation energy transfer between nucleobases of natural DNA. J. Am. Chem. Soc..

[61] Markovitsi D. (2016). UV-induced DNA damage: The role of electronic excited states. Photochem. Photobiol..

[62] Zhang Y., Dood J., Beckstead A.A., Li X.-B., Nguyen K.V., Burrows C.J., Improta R., Kohler B. (2015). Photoinduced electron transfer in DNA: Charge shift dynamics between 8-oxo-guanine anion and adenine. J. Phys. Chem. B.

[63] Zhang Y., Dood J., Beckstead A.A., Li X.-B., Nguyen K.V., Burrows C.J., Improta R., Kohler B. (2014). Efficient UV-induced charge separation and recombination in an 8-oxoguanine-containing dinucleotide. Proc. Natl. Acad. Sci. USA.

[64] Bucher D.B., Schlueter A., Carell T., Zinth W. (2014). Watson-Crick base pairing controls excited-state decay in natural DNA. Angew. Chem. Int. Ed..

[65] Zhang Y., de La Harpe K., Beckstead A.A., Improta R., Kohler B. (2015). UV-induced proton transfer between DNA strands. J. Am. Chem. Soc..

[66] Schultz T., Samoylova E., Radloff W., Hertel I.V., Sobolewski A.L., Domcke W. (2004). Efficient deactivation of a model base pair via excited-state hydrogen transfer. Science.

[67] Kumar A., Sevilla M.D. (2013). Excited state proton-coupled electron transfer in 8-oxoG-C and 8-oxoG-A base pairs: A time dependent density functional theory (TD-DFT) study. Photochem. Photobiol. Sci..

[68] Wang Y., Schlick T. (2007). Distinct energetics and closing pathways for DNA polymerase β with 8-oxog template and different incoming nucleotides. BMC Struct. Biol..

[69] Hsu G.W., Ober M., Carell T., Beese L.S. (2004). Error-Prone replication of oxidatively damaged DNA by a high-fidelity DNA polymerase. Nature.

[70] Cheng K.C., Cahill D.S., Kasai H., Nishimura S., Loeb L.A. (1992). 8-hydroxyguanine, an abundant form of oxidative DNA damage, causes G-T and A-C substitutions. J. Biol. Chem..

[71] Sobolewski A.L., Domcke W. (2007). Computational studies of the photophysics of hydrogen-bonded molecular systems. J. Phys. Chem. A.

[72] Nguyen K.V., Burrows C.J. (2012). Whence flavins? Redox-active ribonucleotides link metabolism and genome repair to the RNA world. Acc. Chem. Res..

[73] Møller C., Plesset M.S. (1934). Note on an approximation treatment for many-electron systems. Phys. Rev..

[74] Dunning T.H. (1989). Gaussian basis sets for use in correlated molecular calculations. I. The atoms boron through neon and hydrogen. J. Chem. Phys..

[75] Schirmer J. (1982). Beyond the random-phase approximation: A new approximation scheme for the polarization propagator. Phys. Rev. A.

[76] Hättig C., Weigend F. (2000). CC2 excitation energy calculations on large molecules using the resolution of the identity approximation. J. Chem. Phys..

[77] (2012). Turbomole, V. 4.

